# Medical News and Items

**Published:** 1859-03

**Authors:** 


### MEDICAL NEWS AND ITEMS.

NATURE AND ART IN DISEASE.—The following remarks are from a very able address by Mr. Browne at the first meeting this session of the Belfast clinical and pathological society:

"There cannot be a question that nature, uninterfered with by drugs and having fair scope for the exercise of her innate sensitive power, will, especially in many acute diseases, overcome the morbid perturbations of the system, restore the normal functions, and bring about that comparative state of the body which we denominate healthy. But how seldom is it that nature has this fair play—this requisite, uninterrupted exercise of the restorative functions! Few, if any of the sick, are so circumstanced that they may not be said to be placed in a condition unsuitable, in many respects, for the wholesome action of the vis medicatrix naturæ, and this may arise from necessity, ignorance, or the pseudo-medical knowledge of the patient and friends.—Take, for instance, a case of measles, scarlet or ordinary fever, and see how much necessity, ignorance, or pseudo-medical skill may complicate the complaint, and retard, if not entirely nullify the efforts of nature to shake off the disease. Then it is that the first display of the curative powers of the medical art comes into operation. The experienced physician at once sees what it is that interferes with the natural progress of the malady. Necessity in one case compels the unhappy patient to be in a low, damp, badly-ventilated situation, without nourishment or without the means of cleanliness; ignorance, in another case, excludes the light and air, and heaps on loads of bed-clothes, and pours down floods of warm drinks; while, in a third case the pseudo-medical knowledge of the patient and friends employs all the domestic remedies, from a teaspoonful of sulphur to a glass of whisky punch, or from the hot posset, drugged with saltpetre, to the cold and nauseous draught of Epsom salts. Now, what are the curative means which the experienced physician first employs in the instances I have just related? He merely vindicates the rights of outraged nature.—He removes the first case into a pure, dry air, administers proper nutriment, gives a cleansing bath, and supplies fresh linen; in the next, he admits a due supply of light, ventilates the apartment, reduces the clothing, forbids the tepid inundations, and allows the use of pure cold water; while, in the third, he employs similar conditions, and strictly excludes every portion of previous domestic drenching. Having done so, he quietly watches the progress of his cases, and, in almost every instance, he finds that he has done all that art requires him to do; nature accomplishes the rest. Yet, in all these instances, tho he never has exhibited a single drug, will any reasonable man say, that by his knowledge of medical science, he has not accomplished all that could have be done—namely, by having put his patients in the right road to recovery, the disease has been cured? Nay, more, has he not thus simply proved the first principles of the curative power of medical art, by his having removed the impediment which obstructed nature in her struggle with disease?"—*Virginia Medical Journal.*

